# Effects of a school-based physical activity intervention on children with intellectual disability: a cluster randomized trial

**DOI:** 10.1186/s12966-025-01798-5

**Published:** 2025-07-25

**Authors:** Michael Noetel, Taren Sanders, Danielle Tracey, David R. Lubans, Viviene A. Temple, Andrew Bennie, James Conigrave, Mark Babic, Bridget Booker, Rebecca Pagano, James Boyer, Chris Lonsdale

**Affiliations:** 1 School of Psychology, the University of Queensland, St Lucia, 4072 Australia; 2 Institute for Positive Psychology and Education, Australian Catholic University, North Sydney, Australia; 3 School of Education, Translational Health Research Institute, Western Sydney University, Sydney, Australia; 4 Centre for Active Living and Learning, University of Newcastle, Callaghan, Newcastle, Australia; 5 School of Exercise Science, Physical and Health Education, University of Victoria, Victoria, Canada; 6 Darug Country, School of Health Sciences, Western Sydney University, Sydney, Australia; 7 Centre for Alcohol Policy Research, La Trobe University, Bundoora, Australia; 8 School of Education, College of Human and Social Futures, University of Newcastle, Callaghan, Newcastle, Australia; 9 School of Education, Australian Catholic University, Strathfield, Australia; 10 Department of Education, Turrella, Australia; 11 Australian Catholic University, North Sydney, Australia

**Keywords:** Physical activity, Fundamental movement skill, Whole-of-school intervention, Intellectual disability, Inclusive education

## Abstract

**Background:**

Children living with disability have poorer health outcomes than their typically developing peers. They are less physically active and at increased risk of chronic disease. Teacher-led, whole-of-school physical activity interventions are promising levers for population-level change, but are seldom tested among children with disability. We aimed to evaluate the effect of a blended teacher-professional learning program (online and in-person) on fundamental movement skills (FMS) and physical activity among children with intellectual disability.

**Methods:**

In this cluster randomized clinical trial, we randomized 20 government-funded primary schools, including 238 consenting students (Grades 2–5; aged 8–11 years at baseline). Ten schools received the blended teacher-professional learning intervention and 10 were assigned as waitlist controls. The professional learning was designed to support teachers as they implemented a whole-of-school intervention designed to enhance FMS and increase physical activity levels. The school-based intervention was mostly online learning, followed by lesson observations and site visits from project staff. Blinded assessors measured FMS competency using the Test of Gross Motor Development-3. Secondary outcomes were self-concept, enjoyment, wellbeing, 300-yard run time, and accelerometer-measured physical activity.

**Results:**

We found no significant group-by-time effects for the primary outcome (FMS competency: b = 1.07 [95% CI -3.70, 5.84], *p* =.658) or any secondary outcomes.

**Conclusions:**

A school-based intervention did not improve FMS competency or physical activity in children with intellectual disability. Results may be attenuated by the COVID-19 pandemic. Alternatively, low intensity teacher-professional learning interventions may not be enough to improve FMS or physical activity among children with intellectual disability.

**Trial registration:**

*Australian New Zealand Clinical Trials Registry* registration number: *ACTRN12620000405910*, registered: 25/03/2020.

**Supplementary Information:**

The online version contains supplementary material available at 10.1186/s12966-025-01798-5.

## Background

Globally, around 12.5% of school-aged children live with moderate-to-severe disability [1]. In high-income countries like Australia, 65% of boys and 54% of girls with disability have intellectual disability, making it the most common disability among school-aged children [2]. People with disabilities often show great resilience and adaptability [3]. But, they also face significant health disparities, including higher rates of cardiovascular disease, respiratory infections, depression, and premature mortality [4, 5]. Those with intellectual disabilities die 20 years earlier than their neurotypical peers [5]. The World Health Organisation (WHO) attributes a large part of this mortality risk to higher rates of noncommunicable diseases among those with disability [6]. It estimates a $10 return for every $1 spent on disability-inclusive prevention of non-communicable diseases [6]. 

A potent lever for preventing non-communicable diseases is increasing physical activity, particularly among children and adolescents [7]. According to the latest physical activity global report card, children and adolescents in most countries fail to meet physical activity guidelines, with a global average fail rate around 70% [8]. The risk of being insufficiently active is higher for children and adolescents with disability [9, 10]. Physical activity also has more immediate benefits for children with disability, increasing physical and psychosocial health indicators [10, 11]. As a result, the WHO recommends the same amount of physical activity for children with and without disability [7]. 

Schools can be potent settings for increasing physical activity among children [12]. One billion children attend school each day, and they spend more time at school than anywhere except home [12]. Whole-of-school physical activity interventions have been shown to increase health and physical activity among typically developing students [12]. However, there is limited research on effective physical activity interventions for children with disability [13, 14]. A systematic review on children with physical disability found only seven studies, all of which were on cerebral palsy, and none of which were in schools [14]. A review on children with intellectual disability found only five studies [13], only one of which was in schools [15]. While that study found no meaningful quantitative improvements [15], one school-based study on adolescents with intellectual disability found significant increases in many health indicators [16]. That study used a small sample and an intensive research protocol, with two, 45-minute sessions delivered each week for nine months by the research staff [16]. Our research aims to see if a more cost-effective delivery model can deliver similar benefits for children with intellectual disability.

Our current study builds upon three projects for children without disability [17–19]. These studies used whole-of-school interventions to increase cardiorespiratory fitness [17, 18], physical activity [17, 19], wellbeing [19], and movement skill competency among children [17]. These interventions focus on teacher professional learning because (a) teachers are the primary providers of physical education and physical activity opportunities in schools, (b) primary school teachers often report low confidence in delivering physical activity, so benefit from training [20], and (c) training them builds sustainable capacity within schools rather than relying on external providers. By applying principles from the Consolidated Framework for Implementation Research [21] to optimize implementation strategies, the intervention could be delivered cost-effectively at approximately 26 US dollars per student [18]. These implementation strategies also enabled many effects to be maintained when scaling-up the intervention to 115 schools [19]. 

In this project, we adapted the intervention to support *all* children, including those with disability. We focused on measuring effects among children with intellectual disability because it was the most common type of disability among school students [2]. We tested the hypothesis that this teacher professional learning intervention increased fundamental movement skill (FMS) competency among those children. These movement skills (e.g., running, jumping, catching, throwing, balancing) are core learning objectives of primary school physical activity [22], and a major component of physical literacy [23]. They are important because—in contrast to a mere snap-shot of a child’s physical activity—movement skills predict long-term physical activity, cardiorespiratory fitness, and weight status in children [24, 25]. Where some measures of physical literacy (e.g., 20-m shuttle run test) lack reliability and validity among children with intellectual disability [26], fundamental movement skills can be reliably and validly assessed [27]. They also predict many health and developmental outcomes in children with intellectual disabilities [27]. However, we also wanted to know if the program increased physical activity, physical fitness, other predictors of physical activity (i.e., enjoyment), and secondary outcomes of activity (i.e., wellbeing, self-concept). As per other studies in with children without disability [17–19], we predicted the intervention would increase these secondary outcomes. Finally, we aimed to conduct qualitative interviews with teachers and principals to better understand implementation barriers and facilitators, consistent with best practice in implementation science. These data were not intended to evaluate effectiveness, but rather to provide context for our quantitative findings and insights for future intervention development. Overall, we aimed to see whether a teacher professional learning intervention could improve the wellbeing, development and physical activity among primary school students with intellectual disability.

## Methods

### Trial design and participants

We received ethical approval from both the Australian Catholic University Human Research Ethics Committee (approval 2019–106 H) and the New South Wales (NSW) Department of Education (State Education Research and Partnerships number 2019289). We used a cluster randomized trial, nesting students within schools. All government primary schools within 3 h of our university were eligible, as long as the school had at least 10 students between Grades 2 and 5 with intellectual disabilities. We excluded Kindergarten and Grade 1, as per our previous studies [17–19], under the assumption they would have more difficulty with our assessments. We did not formally assess the degree of intellectual disability as part of our study procedures—all participating children had been previously identified as having intellectual disability through standardized assessments verified by the education system. In NSW schools, this identification requires formal cognitive assessment with an IQ score below 70 and deficits in adaptive functioning. At consenting schools, all children with intellectual disabilities between those grades were eligible for data collection, with two exceptions. Our measures were not appropriate for students whose disability prevented them from running (e.g., wheelchair users) or those whose comorbid developmental disorders precluded them from responding to verbal questions (e.g., level 2 and 3 Autism Spectrum Disorder). We made one major change to the protocol following registration: we originally scheduled post-test data collection for 12 months after baseline. At this point in time, COVID-19 led the state government to prohibit data collection in schools for high-risk groups, including those with intellectual disabilities. Rather than abandoning data collection, we followed recommendations to maintain trial integrity, where possible [28]. To allow restrictions to abate, we postponed our endpoint data-collection for three school terms (~ 9 months), making our post-test 21 months after baseline. Given this period spanned two school years, we could not meaningfully nest students within teachers for our main analyses.

### Sample size

In typically developing children, a meta-analysis of interventions found a large pooled effect size on overall gross-motor competency [22]. To estimate our effect size, we used the smallest pooled standardized mean difference reported in that meta-analysis (object control = 0.63). We used data from the Department of Education to estimate eligible students per school (~ 20 students meeting criteria in Grades 2–5; conservative 30% consent rate). We also conservatively estimated the sample size using post-test means, instead of a more statistically powerful ANCOVA (described below). We used G*Power 3 to estimate the required sample size to achieve 80% power [29], then increased this value using a design effect [30] (ICC = 0.08 from previous research) [17]. Given the above parameters, we needed 20 schools (10 intervention, 10 control) with 115 students total (5–6 students per school) to reach > 80% power.

### Recruitment, assignment, randomisation, and blinding

We matched schools on school type (regular vs. schools for specific purposes), Index of Community Socio-Educational Advantage (ICSEA), and location (urban vs. remote). Then, an experienced statistician who was not part of the research team used a computer-generated algorithm to randomise matched schools, 1:1 into treatment and control [31]. 

Outcome assessors were blind to allocation. We were unable to blind teachers because they were aware of the training during recruitment for the trial. We did not tell students whether or not their school received the training, however we could not prevent staff from discussing the training with students. Nevertheless, we judged the likelihood of students’ awareness influencing results would be low.

### Intervention

Full details of our intervention are set out in our protocol [32]. We adapted the ‘internet-based Professional Learning to help teachers promote Activity in Youth’ (iPLAY) [18, 19, 33] intervention for children with disability. The iPLAY program helps schools meet state physical activity guidelines: 150 min of physical activity each week, including moderate and vigorous activity. To do this, iPLAY supported teachers to implement:

1. quality physical education and school sport,
2. daily classroom movement breaks (2–3 min, 2–3 times daily),
3. physically active homework,
4. active playground strategies,
5. community physical activity links (e.g., high quality, low-cost, and appropriate community physical activity programs), and.
6. parent engagement (e.g., through regular newsletter content).

We consulted with special education teachers and experts in intellectual disability to identify how iPLAY should best be adapted for this population. To better support children with disability, iPLAY for Inclusion (iPLAY4i) abbreviated some content that was likely less relevant to these schools (e.g., active homework, where many schools for this population had ‘no homework’ policies). Instead we added content on positive behaviour support [34] and universal design for learning [35]. For example, we trained teachers in the TREE framework where teachers introduce variations (to the Teaching style, Rules, Equipment or Environment) [36] that allow all children to experience success during school sport and physical education sessions (see Supplementary File 3 for details).

Our contact with schools occurred over 3.5 school terms (~ 10 months). We designed the intervention so teachers could implement the strategies throughout the whole 21-month study period. Classroom teachers delivered curricular components (e.g., quality PE and school sport, classroom energises) which were built around making classes SAAFE: [37] Supportive, Active, Autonomous, Fair and Enjoyable. Up to three classroom teachers delivered the non-curricular components of the intervention (e.g., active playgrounds). These ‘iPLAY leaders’ also supported other teachers with implementation of curricular components. As per previous studies [33], schools adopted these strategies with support from a two-hour face-to-face workshop, online resources, and visits from an ‘iPLAY mentor’—an experienced physical education teacher employed by the project team. Those visits provided classroom teachers with one lesson observation, and provided leaders with quarterly planning sessions for their non-curricular components. Lesson observations were conducted during periods where COVID-19 lockdowns were lifted, and rescheduled if slated for weeks affected by lockdowns. Quarterly planning sessions proceeded as scheduled—to the degree possible—and were conducted via videoconference or phone if necessary.

Schools allocated to the control condition continued with their usual practice during the intervention period. After post-test data collection, waitlist control schools were offered the opportunity to receive the iPLAY4i intervention.

### Outcomes

All outcomes were measured at baseline and at 21 months post-test.

#### Primary outcome: test of gross motor development-3 (TGMD-3) [38]

The TGMD-3 has been validated for children with developmental disorders, including intellectual disabilities [39–41]. Due to time constraints, we chose the three skills for each subscale that explained the most variance in children with intellectual disabilities (run, gallop, and hop for locomotor skills; one-handed strike, dribble, and kick for object-control skills) [41, 42]. Children were provided with a physical demonstration by a trained research assistant, a practice attempt, and a set of visual prompts shown to increase reliability and validity [39]. Children completed each skill two times and were video-recorded. These research assistants were given detailed standard operating procedures for conducting the assessment, video demonstrations from the makers of the TGMD-3, and were trained by a senior researcher (MN) in the conduct of the assessment. The outcome was coded by a qualified research assistant who was blind to the treatment allocation. Both of the research assistants responsible for coding (MB, BB) had significant experience coding movement skills using the TGMD-3 (> 60 h). We coded 10% of the data in duplicate to assess inter-rater reliability. We pre-registered our decision to use the total score for the two attempts of these six skills.

#### Secondary outcome measures

Measuring cardiorespiratory fitness in children with intellectual disabilities is challenging. Most assessments fail to meet established reliability and validity standards [26]. We measured cardiovascular fitness using the 300-yard run after pilot testing because: we wanted a short, simple measure, given our young sample; and the 300-yard run has been shown to be reliable in children and adolescents with intellectual disabilities [26, 43, 44]. Children were asked to run or walk as fast as they can over 300 yards, timed by a research assistant.

We measured students’ physical activity over seven days using GENEActiv accelerometers, worn on the non-dominant wrist. Wrist-based accelerometry is valid and acceptable for children with intellectual disabilities [45]. Children were instructed to wear the accelerometers continuously for seven days, including during sleep and water-based activities. We used the GGIR package (version 2.1-0; [46]) in R to process the raw accelerometer data, applying the Euclidean norm minus one (ENMO) method with cut-points validated for children [47, 48] to determine time spent in different physical activity intensities (i.e.,. <56.3 mg for sedentary behavior; 56.3-191.6 mg for light physical activity, and > 191.6 mg for moderate-to-vigorous physical activity). We used school bell-times to examine physical activity within school, during breaks, after school, on weekends, and in total.

We asked students about their enjoyment of physical activity [49], their physical self-concept [50, 51], and their satisfaction with life [52]. We used individual administration, where researchers first ask students whether they agree with a statement, and then to what extent. We also included acquiescence checks to ensure that self-report responses were viable. These checks included the items “Do you make all your own clothes and shoes?” and “Where you live, did you choose who lives next door to you?” If participants responded yes to either of these questions they were excluded from the analyses with self-report outcomes. These methods lead to better validity among children with intellectual disability [50]. 

### Statistical analysis

We used R version 4.4.0 for our analyses [53]. In line with intention-to-treat procedures, all available student data was retained and included in mixed model analyses, even in cases where students did not provide data for both timepoints [54]. We used a two-step approach to manage missing data, while using all available information. When a participant missed measurements during a timepoint (e.g., lost their accelerometer, or no weekend data) but completed other measurements during that timepoint (e.g., completed FMS assessments, or had weekday data), we used multiple imputation to impute the missing measurements. Specifically, we used predictive mean matching with the *mice* package [55] to create 20 imputed datasets, filling missing measurements with other available information about that participant from that timepoint. If a participant missed a timepoint entirely (e.g., they left the school before post-test), we did not impute missing data using multiple imputation—we left the participant in the model and used all available data for linear mixed models, which are robust to missing data. We modelled intervention effects using *lme4* [56]. Student scores were nested within schools via a random intercept. Hypothesized intervention effects were tested via interactions between treatment and time.

As pre-registered, we conducted sensitivity analyses to assess whether findings were robust when controlling for demographic variables (i.e., gender, birth country [Australia vs. other], and language spoken at home [English vs. other]). We also conducted a per-protocol analysis by assessing whether completion of the professional learning moderated the effect of the intervention on the primary outcome. Rather than using an arbitrary cut-off, we used learning analytics to identify the percentage of the course that teachers completed at post-test. When students changed teachers between Year 1 and Year 2, we chose the Year 1 teacher completion rate because those teachers spent more time with that student (12 months versus 6–9 months). We assessed whether this percentage explained variance in the effect of the program on their students.

Two exploratory analyses were added after pre-registration. School-based physical activity interventions have typically worked better for boys than for girls [57], including trials of iPLAY among mainstream students [18]. We therefore added a moderation analysis for gender, across all outcomes. Some intervention components may lend themselves better toward acquisition of different fundamental movement skills, so we also conducted a moderation analysis for the subscale (locomotor skills vs. object control) on the primary outcome.

### Qualitative interviews with teachers

We invited all teachers and principals from intervention schools to participate in a brief interview about their experiences of the program. Questions were oriented around the barriers and facilitators they experienced, and the key changes they implemented. We provided staff with three options to participate: face-to-face at their school, Zoom interview with researcher, or asynchronous interview via Phonic.ai. Teachers and principals were offered $50 AUD for a 20–30 min interview. We conducted three rounds of recruitment for these interviews (as many as ethics approval would permit) across 2021 and 2022. During 2021, schools were impacted by COVID-19 restrictions and 2022 was over two years since teachers started the program. As a result, only three teachers consented, and no principals.

## Results

### Recruitment

**Fig. 1:**
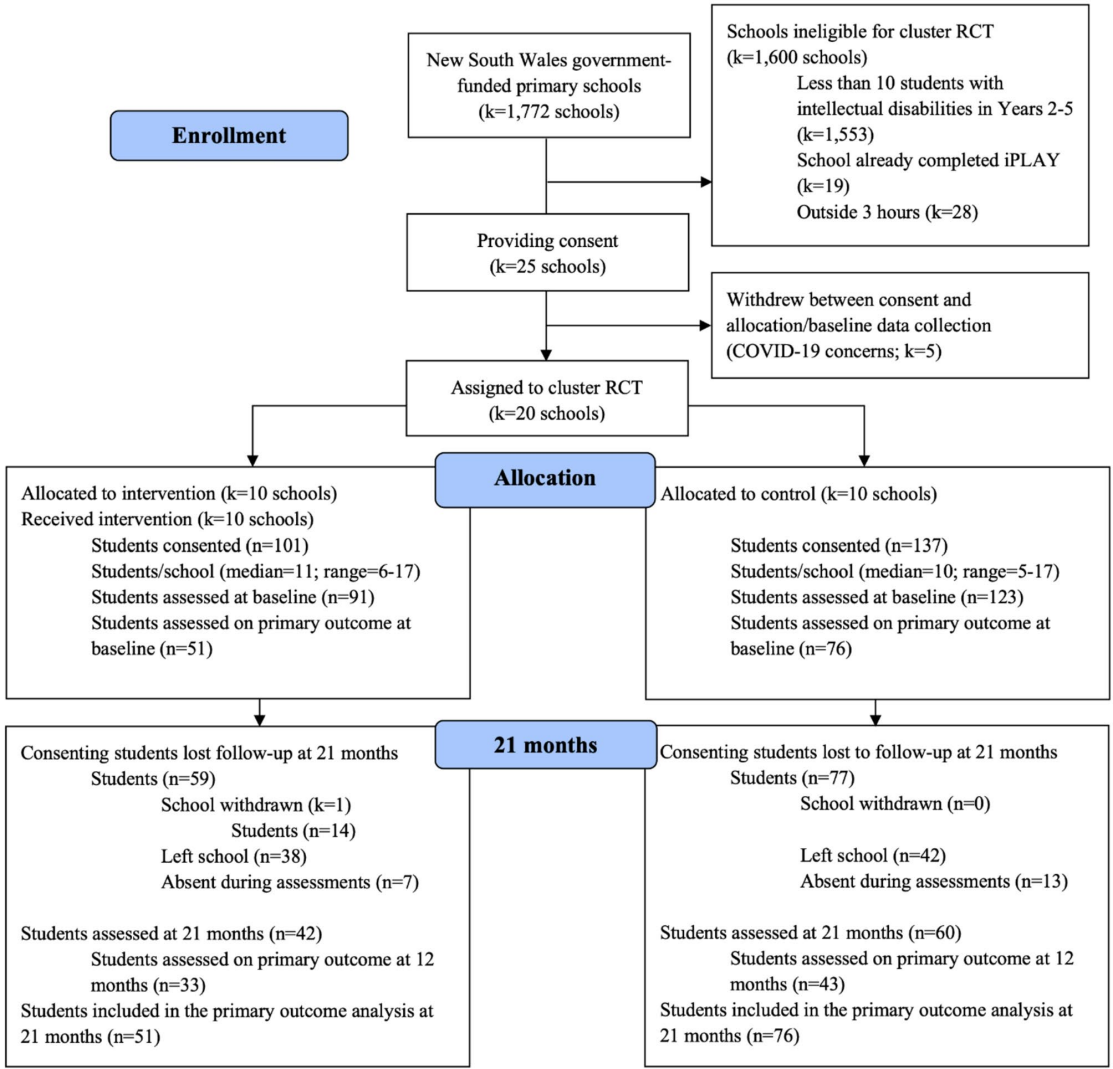
CONSORT flow diagram indicating participant flow throughout the procedure

Recruitment took place between October 2019 and March 2020. Twenty-five schools consented but 5 withdrew before allocation and baseline data collection because of uncertainty around COVID-19. New South Wales schools were instructed to move toward online learning on 23 March 2020 [58]. One school withdrew after baseline data collection and allocation (see Fig. 1). In total, we collected baseline data from 214 students from 20 schools (10 control schools, 10 intervention schools). After 21 months, approximately 33% of participants left participating schools, usually to enter high school.

Participant characteristics were similar across arms (Table 1), with baseline differences only present for self-concept.

**Table 1:** Characteristics of control and intervention arms at baseline

Characteristic	Control	Intervention	*p*-value
Students (n)	123	91	
Schools (n)	10	10	
Female (%)	25.20	28.57	0.746
Australian born (%)	92.68	92.31	0.999
English not spoken at home (%)	10.57	7.69	0.607
Fundamental Movement Skill points	16.14 (9.53)	14.78 (10.93)	0.471
MVPA (mins)	73.39 (30.52)	65.12 (30.46)	0.179
MVPA school (mins)	27.44 (12.90)	23.19 (12.26)	0.082
MVPA school breaks (mins)	12.00 (7.35)	12.00 (7.80)	0.998
MVPA after school (mins)	29.94 (15.81)	24.37 (16.93)	0.106
MVPA weekends (mins)	59.99 (36.28)	54.68 (30.32)	0.382
Self-Concept / 5	4.23 (0.78)	3.89 (0.86)	0.007**
PE Enjoyment / 5	4.51 (0.89)	4.29 (0.90)	0.092
Life Satisfaction / 5	4.52 (0.96)	4.27 (1.14)	0.106

Note. Significance tests were conducted using t-tests for continuous variables, and chi-squared tests for binary variables. ** p <.01

### Primary outcome

**Fig. 2:**
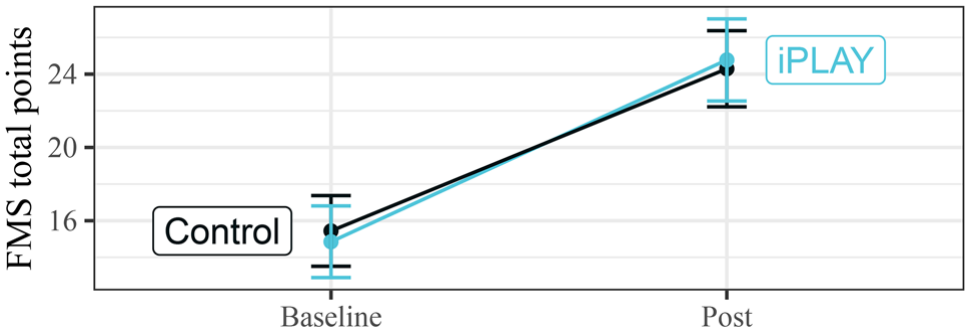
Effect of intervention on fundamental movement skill competency (unadjusted models). *Note*. Error bars show mean ± 1 standard error

There were no statistically significant group-by-time effects of the intervention on fundamental movement skill competency (b = 1.07 [95% CI -3.70, 5.84], *p* =.658; see Fig. 2). This pattern of results was consistent across the unadjusted model, and one which controlled for participant demographics (b = 1.23 [95% CI -3.55, 6.01], *p* =.612; see Supplementary Tables).

**Fig. 3:**
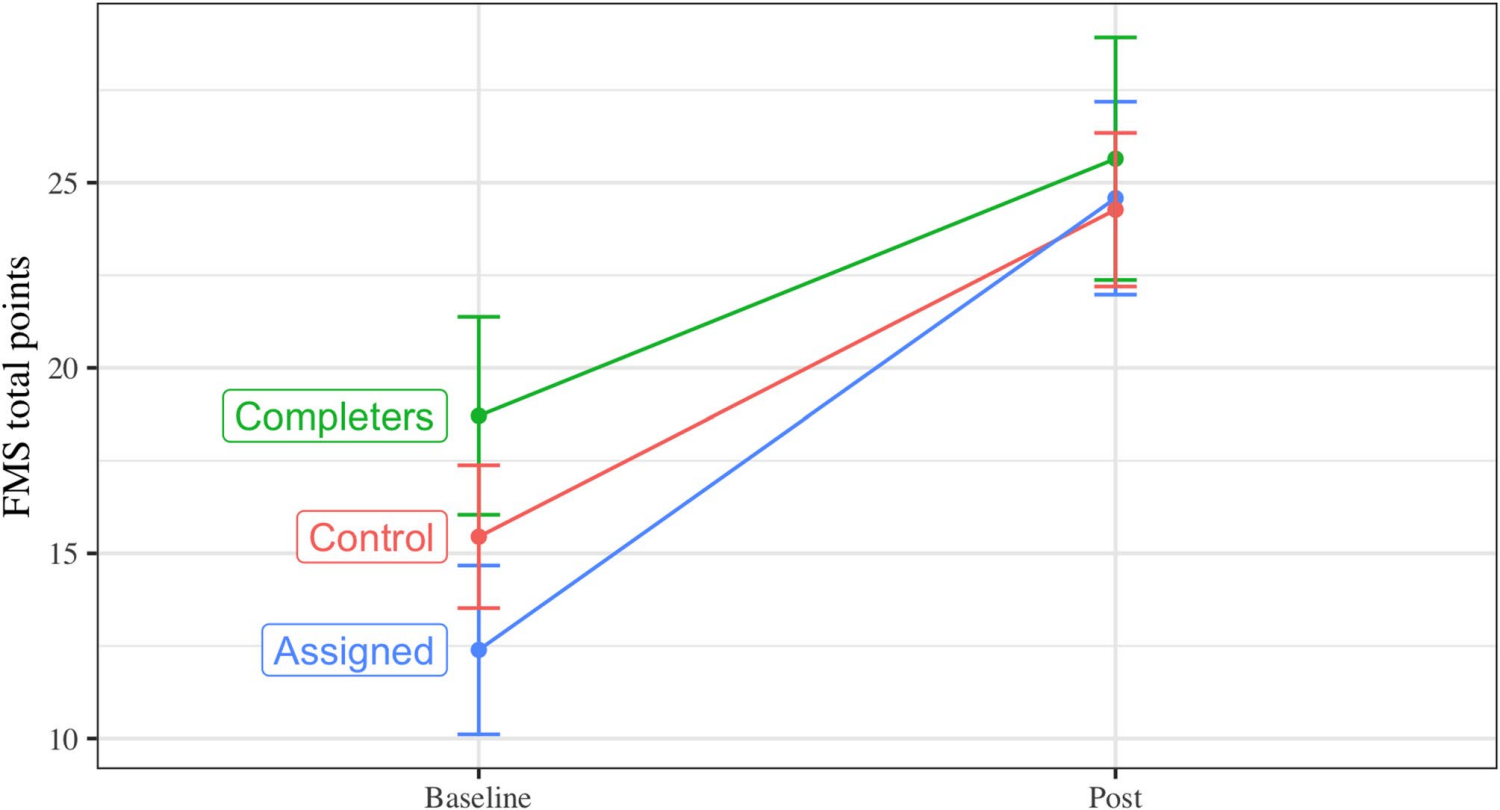
Effect of intervention on fundamental movement skill competency (unadjusted models), with those assigned to the intervention group split by whether or not their teacher completed more than 50% of the program. *Note*. Error bars show mean ± 1 standard error

Per protocol analyses assessed whether intervention effects were explained by the percentage of the course completed by the teachers. Completion rate was bimodal. 45% of teachers did more than half of the online modules—of those, the median completion was 88% of the online course. Of those who did less than half the modules, the median completion was only 18% of the online course. These completion rates significantly predicted students’ FMS competency (b = 10.40 [95% CI 1.94, 18.87], *p* =.016), but did not predict *change* in fundamental movement skills over time (b = 3.81 [95% CI -3.16, 10.79], *p* =.28). As shown in Fig. 3, students of teachers who completed the program had higher FMS at baseline and post, but did not improve faster over time.

### Secondary outcomes

As per Table 2, there were no statistically significant effects on the secondary outcomes of cardiorespiratory fitness (via 300-yard run times, b = 0.10 [95% CI -0.36, 0.56], *p* =.661) student self-concept (b = -0.13 [95% CI -0.62, 0.35], *p* =.589), PE enjoyment (b = -0.04 [95% CI -0.63, 0.56], *p* =.90), life satisfaction (b = -0.28 [95% CI -0.93, 0.38], *p* =.41), or physical activity (e.g., total MVPA b = 8.50 [95% CI -23.83, 40.82], *p* =.60). Results were consistent when controlling for demographic variables (see supplementary materials). All self-report measures had high baseline means (see Table 1) and large negative skews (skewness range = -1.02 to -1.87).

**Table 2:** Effects of intervention on primary and secondary outcomes

Outcome	Intervention effect [95% CI]	SE	t	*p*
Fundamental movement skills	1.07 [-3.70, 5.83]	2.42	0.44	0.659
Run time	0.10 [-0.36, 0.56]	0.23	0.44	0.661
MVPA school time	1.33 [-6.57, 9.23]	3.97	0.34	0.738
MVPA school breaks	-0.65 [-6.01, 4.70]	2.66	-0.25	0.808
MVPA after school	0.76 [-9.41, 10.94]	5.11	0.15	0.882
MVPA weekends	6.92 [-14.06, 27.89]	10.53	0.66	0.513
MVPA all	8.50 [-23.83, 40.82]	16.21	0.52	0.602
Self-concept	-0.13 [-0.62, 0.35]	0.25	-0.54	0.589
PE enjoyment	-0.04 [-0.63, 0.56]	0.30	-0.13	0.900
Life satisfaction	-0.28 [-0.93, 0.38]	0.33	-0.83	0.406

Note. MVPA = Moderate to vigorous physical activity; PE = physical education

### Harms

We had no reports of any students or teachers experiencing harms from their participation in this study.

### Qualitative interviews with teachers

The timing of implementation coincided with significant disruptions to NSW schools: the intervention delivery began in March 2020, just as schools were transitioning to remote learning due to the COVID-19 pandemic. Teachers described how this temporarily impeded their ability to implement the strategies they had learned. Teachers referred to an ‘overwhelming year’ and reported difficulty ‘maintaining momentum’. Other major barriers to adoption were competing priorities placed upon teachers (e.g., curriculum changes, other professional learning). These competing priorities made it hard to prioritise online, self-paced professional learning, unless supported by their principals. Staff found the iPLAY training, mentors, and website helpful (see Supplementary File 2 for quotes). They recommended few changes to the content, if any. Teachers reported focusing their implementation on classroom energizers and differentiation for physical education lessons (e.g., SAAFE principles and TREE framework).

## Discussion

We hypothesized that a blended-learning teacher professional development program would increase fundamental movement skill competency among primary students with intellectual disability. We did not find the intervention increased children’ fundamental movement skills, or any of our secondary outcomes (enjoyment, wellbeing, self-concept, physical activity, or cardiorespiratory fitness). Completion rates and qualitative data suggest adoption was influenced by the COVID-19 pandemic and competing demands placed on teachers. Effects may also have been reduced by the high quality of teaching present in the comparison group.

For fundamental movement skill competency, results suggest effects are stronger from specialized and dedicated instruction, rather than professional learning for generalist or special education teachers. For example, in typically developing children, many interventions have demonstrated an ability to substantially increase fundamental movement skill competency [22, 59]. Like our intervention, these typically involve structured activities focused on skill development, physical activity, and sport [59]. However, the overwhelming majority of those studies were delivered by specialized PE teachers [59], unlike the generalist or special education teachers in our sample. Among children with intellectual disability, exercise programs have demonstrated strong improvements in fundamental movement skills, but few were delivered by regular classroom teachers [60]. Most were delivered outside of class or instead of regular classes. Overall, while exercise and school-based programs can increase fundamental movement skill competency among children, including those with disability, successful interventions might need more training and expertise that we could provide via our intervention model.

The same might be true for our secondary outcomes, like enjoyment, wellbeing, self-concept, cardiorespiratory fitness, or physical activity. Previous research on physical activity interventions for children with intellectual disability generally show small or non-significant effects [13]. Reviews have described the challenges implementing and evaluating programs for children with disability [13, 14]. To evaluate programs, it can be challenging to find measurements that are reliable, valid, and sensitive to change. For example, our measures of self-concept, subjective well being, and PE enjoyment all demonstrated possible ceiling effects, with baseline means near the maximum possible score. For interventions, programs also often need specialized training, higher customisation, and higher staff-to-student ratios than would be needed for typically developing students. Programs that have been effective for those with disability appear to have significantly higher costs per participant (e.g., modified bikes, intensive programs, external staff) [13]. Our program was less intensive than other school-based physical activity interventions for children with disability (with, for example, 70 + sessions exercise sessions facilitated by research staff) [16]. It is likely train-the-trainer models like ours have smaller effect sizes than direct interventions by researchers [61]. Among typically developing students, low-cost train-the-trainer physical activity interventions have been delivered at scale [18, 19], but they also typically demonstrate modest effects (significant only among some students) [57]. Our study was likely underpowered to detect modest effects. Given we saw few benefits for physical activity or fundamental movement skills, it was to be expected that we also saw no change in downstream outcomes like subjective wellbeing, self-concept, or cardiorespiratory fitness.

The data suggest a few other reasons why we may have found no significant benefits. First, around half of teachers who started the program finished the modules. The bimodal distribution may be explained by the incentive structure, where teachers received government-mandated professional learning ‘hours’ for completing the program, but received no hours for doing less. That incentive would explain a bimodal distribution: teachers who needed those hours may have engaged, and others may have already met their quota for hours. It reflects that adoption rates as high as 50% may not generalise to school contexts without these incentives. Alternatively, the bimodal distribution may suggest selection bias in teachers who complete the program. Our data showed teachers who completed the program tended to have students who were more competent at baseline. It is possible that teachers who completed the training are the ones already more engaged in promoting physical activity, or ones teaching students with less severe disability. We do not have data regarding severity of student disability or teacher attitudes toward physical activity, so identifying causes of—and solutions to—this selection bias are important for future research.

Adoption rates like ours are common in the online delivery of professional learning [62]. Engagement in online professional learning is a known challenge, so we tried to embed many of the recommendations for improving engagement from Lee and colleagues (e.g., emphasising intrinsic value, ensuring user friendliness, asking principals to allocate time) [62]. Nevertheless, our study may reveal a trade off between low-cost interventions with low researcher contact time, and high-touch interventions with higher adoption and effect sizes. Future studies may want to compare the cost-effectiveness of our blended learning model against more intensive models with more synchronous time with teachers, to see if this increases adoption.

Our adoption rate may also be partially explained by COVID-19. Intervention delivery began in 2020, a year of lockdowns and remote schooling. Our qualitative data suggested the biggest barriers for teachers were not in the implementation of the iPLAY4i strategies, but that competing demands got in the way of online professional learning. When iPLAY was delivered to regular teachers prior to the pandemic, the same course and incentive structure led to higher completion rates (63% vs. 45% here) [18]. So, if this study were replicated, we anticipate adoption to be higher without the interference of a global pandemic.

Effects may have also been modest due to strong improvements even among the control group. As shown in Fig. 3, even the control group had a 50% improvement in performance on the TGMD-3, relative to baseline. This may be expected given there was 21 months of development between baseline and follow-up. This natural development may have made it more difficult to detect intervention effects, particularly with the extended follow-up necessitated by COVID-19 restrictions. However, as shown in Table 1, the average child was getting over 60 min of moderate-to-vigorous physical activity each school day at baseline, meeting WHO guidelines [7]. Combined with the FMS result, these data suggest children in our sample may be doing well compared against guidelines and benchmarks. These data could represent participant reactivity, where children, teachers, or parents increased physical activity due to the mere process of data collection. Alternatively, these data suggest ‘business as usual’ in these schools may be sufficient to help children with intellectual disabilities at this age. It appears physical activity most precipitously declines somewhat later in development among this population. For example, Shields et al. [63] found that differences in activity between children with and without disabilities become more pronounced in adolescence. Interventions may therefore be more important as students start transitioning into adolescence [64]. Whole-of-school interventions may also need to target different components to what we included. Our curricular components often focused on adapting lessons to children’s abilities (e.g., via the TREE framework) [36]. This kind of differentiation may be routine for teachers of students with disability; if this kind of adaptation is already routine, training may not be valuable. In contrast, the small amount of qualitative data we had suggested teachers got value from the classroom energizers. It is possible that our intervention could have focused less on improving the quality of physical education, but instead focused on increasing the *opportunity* for students to be active (e.g., via more frequent, brief physical activity sessions) [64]. Future studies should aim to explore current teacher beliefs and knowledge gaps before and after the intervention. Doing so would help assess what teachers are learning most from the professional learning interventions, and what learning best predicts student outcomes.

### Limitations and future directions

Our results have a number of important caveats. First, our study was significantly impacted by COVID-19. While it was important for clinical trials to continue despite the pandemic [28], government-imposed restrictions meant we needed to delay end-point data collection by three school terms. This delay may have allowed some intervention effects to wash out. We did not see effects wash out in our interventions for children without disability [17–19], even after 24 months, likely because the intervention built the capacity of teachers who continued to work with students. However, it is possible that there is higher variability in student trajectories among this population, meaning the delayed end-point reduced our sensitivity to identify intervention effects. Unfortunately, we do not have data to test this assumption. The delayed end-point also meant over 30% of our sample left for high school and were lost to follow-up. With this dropout—and a large number of participants who could not, or would not complete our primary outcome—our study was likely underpowered. It also appears our intervention effects were smaller than those from meta-analyses of fundamental movement skill training for typically developing children [22]. Therefore, while our intervention was low cost and therefore easy to scale, the effects may be smaller than more intensive interventions for students without disability. We also tried to target a range of physical activity outcomes, not only fundamental movement skills. Teachers were encouraged to make physical activity more motivating, more frequent, and better differentiated to student abilities. It is likely that an intervention would have demonstrated stronger effects for our primary outcome if it focused *only* on fundamental movement skills. Future studies may want to compare the outcomes from focused interventions against a diffuse intervention like ours, targeting a range of outcomes.

Our primary outcome (fundamental movement skill competency) had blinded, trained researchers using a measure validated among children with intellectual disabilities (TGMD-3) [39–41]. However, many of our other measures may have less good reliability or validity. For example, our self-report measures (e.g., self-concept, subjective wellbeing) were drawn from validated measures, but the response scales might be less sensitive to changes than measures appropriate for typically developing children. This may explain why we found no changes on self-reported measures, but previous evaluations of iPLAY among typically developing students saw gains in wellbeing, motivation, and enjoyment [19]. Alternatively, children with disability may have a greater number of factors that influence these variables (e.g., direct and indirect consequences of their disability). So, against these background factors, the effects from our professional learning program may be too small to detect. We did not conduct power analyses for these secondary outcomes. Future studies may want to better estimate the influence of physical activity on, say, wellbeing and adequately power their design to detect these effects.

## Conclusions

Our study found this school based intervention did not significantly increase fundamental movement skills, physical activity, or self-reported outcomes among children with intellectual disability. Effective, scalable school-based physical activity interventions for children with disability remain elusive. Given the percentage of the population with disability, and increased risk of chronic disease among this population, researchers should continue exploring novel ways of increasing physical activity and physical literacy among this population.

## Electronic supplementary material

Below is the link to the electronic supplementary material.

Supplementary Material 1

Supplementary Material 2

Supplementary Material 3

## Data Availability

Deidentified data and code for reproducing the analyses are available on the Open Science Framework: https://osf.io/jqt32.

## Abbreviations

FMS: Fundamental movement skills
iPLAY: Internet-based Professional Learning to help teachers promote Activity in Youth
iPLAY4i: iPLAY for Inclusion
MVPA: Moderate to vigorous physical activity
NSW: New South Wales
PE: Physical education
SAAFE: Supportive, Active, Autonomous, Fair and Enjoyable
TGMD-3: Test of Gross Motor Development-3
TREE: Teaching style, Rules, Equipment or Environment
WHO: World Health Organisation
