# Supplementary material for: Effects of a school-based physical activity intervention on children with intellectual disability: a cluster randomized trial
Source: Int J Behav Nutr Phys Act. 2025 Jul 25;22:103. doi: 10.1186/s12966-025-01798-5 (PMC12291417; doi:10.1186/s12966-025-01798-5)
Supplement: Supplementary file 3 — Supplementary Material 3 [file 12966_2025_1798_MOESM3_ESM.docx]

# Table of Contents

[Supplementary File 1: Supplementary Analysis Tables](#_9wtracgxc1c7)

[Analysis for Boys Only](#_sb8euuhisc9t)

[Primary Outcome](#_8gdkl11wsa3r)

[Secondary Outcomes](#_hv02fen0jewz)

[Analysis for Girls Only](#_3k0qe3imvoic)

[Primary Outcome](#_o2oxre7xjs2)

[Secondary Outcomes](#_ueynru5zcnsg)

[Primary Outcome by Subscale (Locomotor and Object Control)](#_4k8ht779l12f)

[Supplementary File 2: Qualitative Analysis of Teacher Interviews](#_32uaofuvae05)

[Method](#_59yvqodjplbq)

[Results](#_nrr2uqu92l6n)

[Motivation for Participation. Teachers described two primary motivations for participating in the program: professional development and school-wide initiatives.](#_f5gkoypt4yhu)

[Implementation Barriers. Teachers identified several barriers to implementation, particularly related to competing priorities and time constraints.](#_wqihinpsjg0v)

[Perceived Benefits. Teachers identified multiple benefits of the program for schools, teachers, and students.](#_gazqvoix8lc4)

[Support Structures. Teachers emphasised the importance of mentorship and leadership support in successful implementation.](#_px69yetyu5y9)

[Program Resources. Teachers valued the program's online platform and resources.](#_qa1yctexqqs6)

[Implementation Strategies. Teachers described various strategies for successful implementation.](#_lgdddfftpqmc)

[Teaching Practice Changes. Teachers reported significant changes in their teaching practice after implementing iPLAY4i.](#_9u2u9hynuuvo)

[Supplementary File 3: Comparison of Curricula for iPLAY versus iPLAY for Inclusion (iPLAY4i)](#_ityotpczbfus)

[Theoretical Framework Additions](#_4oq4val9axvd)

[Module Structure Changes](#_2c7qz4ckryn2)

[Added Module](#_azd7uho3cy4b)

[Module Content Modifications](#_19ij1rhamluy)

[Content Removed](#_kr3re9fe21pf)

[Content Added](#_t1sthf90ze2q)

[Key Content Adaptations Across All Modules](#_z8ue89evts26)

[Abbreviated Content](#_78zjbupqvfkv)

[Expanded Content](#_9e2yisj5qku4)

#

# Supplementary File 1: Supplementary Analysis Tables

##### Table S1 *The effect of the intervention on fundamental movement skills, after adjusting for covariates*

|  | *b [95% CI]* | *se* | *t* | *p* |
| --- | --- | --- | --- | --- |
| (Intercept) | 13.96 [6.57, 21.35] | 3.74 | 3.73 | < .001 |
| Intervention group | -0.62 [-6.11, 4.87] | 2.79 | -0.22 | .824 |
| Effect of time | 8.82 [5.66, 11.99] | 1.60 | 5.50 | < .001 |
| Female | -1.89 [-4.82, 1.04] | 1.49 | -1.27 | .206 |
| Born Aus | 1.96 [-4.46, 8.39] | 3.25 | 0.60 | .547 |
| Non-english speaking | 1.60 [-3.61, 6.81] | 2.63 | 0.61 | .544 |
| Effect of intervention | 1.23 [-3.55, 6.01] | 2.42 | 0.51 | .612 |

*Note.* The term ‘Effect of intervention’ shows the additional improvement the intervention arm had, relative to control.

## Analysis for Boys Only

### Primary Outcome

##### Table S2 *The effect of the intervention on fundamental movement skills — data filtered to only include boys*

|  | *b [95% CI]* | *se* | *t* | *p* |
| --- | --- | --- | --- | --- |
| (Intercept) | 15.57 [11.37, 19.76] | 2.12 | 7.33 | < .001 |
| Intervention Group | -0.41 [-6.43, 5.61] | 3.05 | -0.14 | .893 |
| Effect of time | 8.46 [4.48, 12.44] | 2.01 | 4.21 | < .001 |
| Effect of Intervention | 3.69 [-2.49, 9.87] | 3.13 | 1.18 | .240 |

*Note.* The term ‘Effect of intervention’ shows the additional improvement the intervention arm had, relative to control.

### Secondary Outcomes

##### Table S3 *The impact of the intervention on secondary outcomes — data filtered to only include boys*

|  | *b [95% CI]* | *se* | *t* | *p* |
| --- | --- | --- | --- | --- |
| **Cardiorespiratory fitness** |  |  |  |  |
| (Intercept) | 2.09 [1.76, 2.43] | 0.17 | 12.36 | < .001 |
| Intervention Group | 0.05 [-0.40, 0.50] | 0.23 | 0.22 | .827 |
| Effect of time | 0.01 [-0.46, 0.48] | 0.23 | 0.06 | .954 |
| Effect of Intervention | 0.06 [-0.56, 0.67] | 0.31 | 0.18 | .855 |
| **Self concept** |  |  |  |  |
| (Intercept) | 4.16 [3.91, 4.41] | 0.13 | 33.00 | < .001 |
| Intervention Group | -0.15 [-0.52, 0.22] | 0.19 | -0.79 | .432 |
| Effect of time | 0.04 [-0.33, 0.42] | 0.19 | 0.23 | .819 |
| Effect of Intervention | -0.29 [-0.85, 0.27] | 0.28 | -1.02 | .311 |
| **PE enjoyment** |  |  |  |  |
| (Intercept) | 4.46 [4.18, 4.74] | 0.14 | 31.12 | < .001 |
| Intervention Group | -0.20 [-0.62, 0.22] | 0.21 | -0.94 | .348 |
| Effect of time | -0.01 [-0.44, 0.42] | 0.22 | -0.04 | .967 |
| Effect of Intervention | -0.08 [-0.72, 0.57] | 0.33 | -0.24 | .810 |
| **Life satisfaction** |  |  |  |  |
| (Intercept) | 4.67 [4.36, 4.98] | 0.16 | 29.81 | < .001 |
| Intervention Group | -0.53 [-0.99, -0.07] | 0.23 | -2.26 | .026 |
| Effect of time | -0.12 [-0.68, 0.45] | 0.28 | -0.41 | .680 |
| Effect of Intervention | -0.13 [-0.95, 0.70] | 0.42 | -0.31 | .760 |

##

##### Table S4 *The effect of the intervention on physical activity — data filtered to only include boys*

|  | *b [95% CI]* | *se* | *t* | *p* |
| --- | --- | --- | --- | --- |
| **MVPA at School** |  |  |  |  |
| (Intercept) | 27.67 [23.65, 31.69] | 2.03 | 13.65 | < .001 |
| Intervention Group | -2.84 [-8.65, 2.97] | 2.93 | -0.97 | .336 |
| Effect of time | -2.88 [-9.80, 4.04] | 3.45 | -0.83 | .408 |
| Effect of Intervention | 1.31 [-8.94, 11.55] | 5.13 | 0.25 | .800 |
| **MVPA Recess and Lunch** |  |  |  |  |
| (Intercept) | 11.79 [9.00, 14.58] | 1.39 | 8.49 | < .001 |
| Intervention Group | 0.29 [-3.65, 4.24] | 1.97 | 0.15 | .881 |
| Effect of time | -1.12 [-5.13, 2.89] | 1.99 | -0.56 | .578 |
| Effect of Intervention | -1.06 [-7.04, 4.91] | 2.98 | -0.36 | .723 |
| **MVPA After School** |  |  |  |  |
| (Intercept) | 27.52 [21.54, 33.51] | 2.97 | 9.25 | < .001 |
| Intervention Group | -2.78 [-10.82, 5.26] | 4.03 | -0.69 | .493 |
| Effect of time | -4.00 [-13.84, 5.84] | 4.84 | -0.83 | .414 |
| Effect of Intervention | 2.90 [-11.51, 17.32] | 7.14 | 0.41 | .686 |
| **MVPA on Weekend** |  |  |  |  |
| (Intercept) | 55.50 [44.92, 66.07] | 5.34 | 10.40 | < .001 |
| Intervention Group | 0.76 [-15.06, 16.57] | 7.97 | 0.09 | .925 |
| Effect of time | -6.31 [-23.24, 10.61] | 8.46 | -0.75 | .458 |
| Effect of Intervention | 6.63 [-21.33, 34.59] | 13.93 | 0.48 | .636 |
| **Total MVPA** |  |  |  |  |
| (Intercept) | 63.66 [55.59, 71.74] | 4.05 | 15.70 | < .001 |
| Intervention Group | -3.64 [-15.51, 8.22] | 5.96 | -0.61 | .543 |
| Effect of time | -7.59 [-20.48, 5.29] | 6.41 | -1.18 | .242 |
| Effect of Intervention | 4.22 [-14.21, 22.65] | 9.24 | 0.46 | .649 |

## Analysis for Girls Only

### Primary Outcome

##### Table S5 *The effect of the intervention on fundamental movement skills — data filtered to only include girls*

|  | b [95% CI] | se | t | p |
| --- | --- | --- | --- | --- |
| (Intercept) | 16.69 [12.54, 20.83] | 2.07 | 8.05 | < .001 |
| Intervention Group | -3.68 [-9.94, 2.57] | 3.12 | -1.18 | .243 |
| Effect of time | 9.42 [4.58, 14.26] | 2.40 | 3.92 | < .001 |
| Effect of Intervention | -2.70 [-9.98, 4.58] | 3.60 | -0.75 | .458 |

*Note.* The term ‘Effect of intervention’ shows the additional improvement the intervention arm had, relative to control.

### Secondary Outcomes

##### Table S6 The impact of the intervention on secondary outcomes — data filtered to only include girls

|  | b [95% CI] | se | t | p |
| --- | --- | --- | --- | --- |
| **Cardiorespiratory fitness** |  |  |  |  |
| (Intercept) | 2.10 [1.73, 2.47] | 0.18 | 11.47 | < .001 |
| Intervention Group | 0.03 [-0.50, 0.56] | 0.26 | 0.11 | .910 |
| Effect of time | 0.20 [-0.42, 0.82] | 0.30 | 0.68 | .506 |
| Effect of Intervention | 0.00 [-0.78, 0.78] | 0.38 | -0.01 | .995 |
| **Self concept** |  |  |  |  |
| (Intercept) | 3.89 [3.53, 4.25] | 0.18 | 21.50 | < .001 |
| Intervention Group | -0.04 [-0.70, 0.62] | 0.33 | -0.13 | .900 |
| Effect of time | -0.34 [-0.97, 0.29] | 0.31 | -1.08 | .286 |
| Effect of Intervention | 0.04 [-0.96, 1.03] | 0.49 | 0.07 | .943 |
| **PE enjoyment** |  |  |  |  |
| (Intercept) | 4.39 [3.93, 4.84] | 0.23 | 19.18 | < .001 |
| Intervention Group | -0.16 [-0.97, 0.66] | 0.40 | -0.39 | .696 |
| Effect of time | -0.02 [-0.81, 0.77] | 0.39 | -0.05 | .962 |
| Effect of Intervention | 0.13 [-1.16, 1.43] | 0.64 | 0.21 | .837 |
| **Life satisfaction** |  |  |  |  |
| (Intercept) | 4.42 [3.99, 4.84] | 0.21 | 21.02 | < .001 |
| Intervention Group | -0.09 [-0.84, 0.66] | 0.37 | -0.24 | .815 |
| Effect of time | 0.24 [-0.54, 1.02] | 0.39 | 0.62 | .542 |
| Effect of Intervention | -0.58 [-1.80, 0.63] | 0.60 | -0.97 | .337 |

##### Table S7 *The effect of the intervention on physical activity — data filtered to only include girls.*

|  | *b [95% CI]* | *se* | *t* | *p* |
| --- | --- | --- | --- | --- |
| **MVPA at School** |  |  |  |  |
| (Intercept) | 24.94 [19.52, 30.36] | 2.69 | 9.27 | < .001 |
| Intervention Group | -5.16 [-13.02, 2.71] | 3.91 | -1.32 | .193 |
| Effect of time | -5.24 [-16.09, 5.62] | 5.16 | -1.01 | .324 |
| Effect of Intervention | 5.38 [-8.35, 19.12] | 6.65 | 0.81 | .426 |
| MVPA R and L |  |  |  |  |
| (Intercept) | 12.39 [9.50, 15.27] | 1.43 | 8.66 | < .001 |
| Intervention Group | -1.64 [-6.05, 2.77] | 2.18 | -0.75 | .456 |
| Effect of time | -2.85 [-7.17, 1.47] | 2.14 | -1.33 | .190 |
| Effect of Intervention | 1.61 [-5.15, 8.38] | 3.33 | 0.49 | .631 |
| MVPA After School |  |  |  |  |
| (Intercept) | 26.22 [18.83, 33.61] | 3.67 | 7.15 | < .001 |
| Intervention Group | -1.60 [-13.14, 9.95] | 5.71 | -0.28 | .781 |
| Effect of time | -2.99 [-17.82, 11.84] | 7.09 | -0.42 | .678 |
| Effect of Intervention | 1.27 [-21.59, 24.13] | 10.83 | 0.12 | .908 |
| MVPA on Weekend |  |  |  |  |
| (Intercept) | 57.28 [42.27, 72.29] | 7.46 | 7.68 | < .001 |
| Intervention Group | -7.76 [-30.20, 14.67] | 11.15 | -0.70 | .490 |
| Effect of time | -10.46 [-36.65, 15.74] | 12.74 | -0.82 | .419 |
| Effect of Intervention | 7.92 [-28.35, 44.19] | 17.72 | 0.45 | .658 |
| Total MVPA |  |  |  |  |
| (Intercept) | 61.71 [50.86, 72.56] | 5.41 | 11.42 | < .001 |
| Intervention Group | -8.22 [-24.40, 7.95] | 8.06 | -1.02 | .312 |
| Effect of time | -10.77 [-31.07, 9.54] | 9.78 | -1.10 | .283 |
| Effect of Intervention | 7.89 [-21.54, 37.33] | 14.17 | 0.56 | .583 |

## Primary Outcome by Subscale (Locomotor and Object Control)

In addition to ‘Total fundamental Movement Skill’ score, we also decomposed our main findings by subcale (skill grouping). That is, we looked at the effect of the intervention on locomotor and object control.

##### Table S8 *The effect of the intervention on Fundamental Movement Skills by subscale*

|  | *b [95% CI]* | *se* | *t* | *p* |
| --- | --- | --- | --- | --- |
| **Locomotion** |  |  |  |  |
| (Intercept) | 7.98 [5.99, 9.97] | 1.01 | 7.91 | < .001 |
| Intervention group | 0.03 [-2.85, 2.91] | 1.46 | 0.02 | .982 |
| Effect of time | 4.21 [2.27, 6.16] | 0.98 | 4.28 | < .001 |
| Effect of intervention | -0.23 [-3.16, 2.71] | 1.49 | -0.15 | .879 |
| **Object Control** |  |  |  |  |
| (Intercept) | 7.50 [5.32, 9.69] | 1.11 | 6.77 | < .001 |
| Intervention group | -0.64 [-3.78, 2.50] | 1.59 | -0.40 | .688 |
| Effect of time | 4.59 [2.86, 6.32] | 0.88 | 5.24 | < .001 |
| Effect of intervention | 1.32 [-1.39, 4.03] | 1.37 | 0.96 | .339 |

*Note.* The term ‘Effect of intervention’ shows the additional improvement the intervention arm had, relative to control.

# Supplementary File 2: Qualitative Analysis of Teacher Interviews

All names and school identifiers have been replaced with randomly generated alternatives to protect participant confidentiality.

## Method

Individual interviews were conducted with three teachers who implemented the iPLAY4i program in their schools. Interviews explored teachers' experiences with the program, including motivations, barriers, benefits, and implementation strategies. Data were analysed using thematic analysis. Quotes have been minimally edited for clarity while maintaining the participants' intended meaning.

## Results

Analysis yielded seven primary themes related to teachers' experiences implementing iPLAY4i:

1. motivation for participation;
2. implementation barriers;
3. perceived benefits;
4. support structures;
5. program resources;
6. implementation strategies; and
7. teaching practice changes.

### Motivation for Participation. Teachers described two primary motivations for participating in the program: professional development and school-wide initiatives.

#### Professional Development

"I decided to do the iPLAY4i training to build my capacity to teach and implement PE and sport programmes across the school." (Participant 1)

#### School-Wide Initiative

"Our iPLAY training that we did at school was actually part of our professional development plan as a whole school. So that's the reason why we did the iPLAY training." (Participant 2)

### Implementation Barriers. Teachers identified several barriers to implementation, particularly related to COVID-19, competing priorities and time constraints.

####

#### COVID-19 Disruptions

"We have all these amazing ideas. I'm looking forward to it... but... for the record, we did most of our training in term two and ready to go for term three, and then lockdown happened and we haven't been able to do any of that yet."

#### Competing Priorities

"Some of the major barriers in completing the iPLAY4i training were really based around the time it took to set aside, really amongst a very busy school calendar. With ever-growing PL teacher requirements at the school, trying to really keep the iPLAY at the forefront was a struggle." (Participant 1)

"Because of the curriculum change over this year, we had to actually train up getting ready for next year. So that actually took up quite a bit of our time. And the iPLAY training was taking up quite a bit of our time as well." (Participant 2)

#### Time Management Challenges

"It really does require teachers to be self-driven and self-directed. And, you know, as we know, time can certainly get away." (Participant 1)

"It's just basically finding the time to do the training modules... I suppose maybe this particular year was quite overwhelming with everything that we had to get done." (Participant 2)

#### Varying Teacher Adoption

"Some of those teachers in the unit are really excited. One's a big yoga fanatic, really into it. The other was really young and super energetic, and wanted to go and do all these things…There's another two that are a little bit older and a bit set in their ways and not wanting to change things. You can see they're a bit reluctant, but I think they'll see the benefits of it." (Participant 3)

### Perceived Benefits. Teachers identified multiple benefits of the program for schools, teachers, and students.

#### Benefits for Schools

"It certainly benefits schools and students reaching their learning outcomes. By having regular play breaks and regular iPLAY sessions, classroom energisers and that sort of thing, the kids are able to concentrate and focus for a longer period of time. I think it builds a positive environment and school culture within the school as well." (Participant 1)

"It got all the students active because we did it as a whole school. All the teachers were involved, all the students were involved." (Participant 2)

#### Benefits for Teachers

"It does actually build our capacity to teach sport and PE more effectively, and also to ensure that those students who do have special needs aren't forgotten about and they are included in teaching them those fundamental movement skills." (Participant 1)

"The way that the iPLAY is set up with all the instructions, you've got the videos that we can show the students how to do movements, the fine motor skills, all that sort of stuff... The way that it was set up benefits the teachers greatly, to help teach the students how to do the right actions." (Participant 2)

#### Benefits for Students with Intellectual Disabilities

"They also see that their students in the mainstream are also doing iPLAY. So there's no separation there, fully included and fully immersed in the iPLAY activities." (Participant 1)

"Having a lot of the games set up so that there's no winners and there's no losers and the games being able to be modified to suit the individual needs, that was a great benefit for the students. They got out there. They enjoyed it." (Participant 2)

#### Behaviour Management Benefits

“Stage Three were going out for an extra hour every day because behaviors were really skyrocketing, negative behaviors, and so it was sort of mandated by the principal that Stage Three just had to move more.”

### Support Structures. Teachers emphasised the importance of mentorship and leadership support in successful implementation.

#### Mentor Support

"I found the help given by my mentor was outstanding. He comes with a wealth of experience, interest and passion for physical activity, for sport, for inclusivity. And he's very open to seeing the different setups in the school. He was very open to coming into our support unit. He didn't have any judgments." (Participant 1)

"The workshop David came out and did with us was actually quite fun. I really enjoyed that. And that gave me great insight on different things that I could do with my students." (Participant 2)

#### Leadership Support

"It did help that I was an iPLAY leader, so I was having regular meetings and prompts from my iPLAY team mentor to keep me on track in terms of completing the training. We also have a supportive school principal who did actually allocate some extra PL time for teachers so that we could complete that training." (Participant 1)

"Karen, our team leader for the iPLAY, kept tabs on us and just made sure that we were staying on track to get our training done. So she was a great help with that as well." (Participant 2)

### Program Resources. Teachers valued the program's online platform and resources.

#### Website and Resources

"The iPLAY website is fantastic. Having the instructional videos, the resources, the lesson plans, the action plans, even the assessment sheets to download—that's just brilliant and it really helps me create a solid understanding of my students." (Participant 1)

"The iPLAY website is fantastic. I loved it. It's well set out. With the resources I loved that page because there are so many resources I could not believe. And the way that it's structured as well, where you've got your resource category, you've got your stages, your warm-up activities, your fundamental movement skills... you can just pick and choose what you're wanting to do with the students." (Participant 2)

### Implementation Strategies. Teachers described various strategies for successful implementation.

#### Starting Small

"I sort of made the decision to start small, just start with some energiser breaks and just really seeing the kids and how engaged they were. And then they start asking and they keep saying 'Miss, we haven't done iPLAY today,' and so it really comes from them, too." (Participant 1)

#### Adaptations for Support Unit

"We printed off some of the sporting activities, the simple ones, the ones that were more suited to our students. We just printed off a few of those, laminated them and just grabbed one every now and then when we went out and did little iPLAY activities with the students." (Participant 2)

"I had to do a lot of adjustments during my lesson observation to get my students involved. I did use visuals, and I had to give them a lot of instructions beforehand to prep them to get ready for the actual sporting activities when we went out." (Participant 2)

### Teaching Practice Changes. Teachers reported significant changes in their teaching practice after implementing iPLAY4i.

#### Increased Confidence

"It has really given me the confidence to say that I can do it because I was always the first person to say, 'Oh, I'm not a sporty teacher, my area of expertise is more the arts or English.' And now that I've done the iPLAY and I've realised and experienced how easy it is to do, and I've seen the effects on the students, I feel really confident, and I approach it in a really positive can-do kind of way." (Participant 1)

#### Application of Frameworks

"Certainly the SAFE—supportive, active, fair and enjoyable—acronym is very helpful, and it's good to fall back on those principles when you feel like you can't do it or you can't understand it." (Participant 1)

"We always consider the SAFE principles. And we always look at the TREE framework when we're doing anything with our students." (Participant 2)

#### Implementation of Energisers

"I use the energiser breaks in the classroom. Again, I teach students with special needs and more of the mild intellectual disability range. So I have students with ADHD and ODD and autism and dyslexia. So these types of energiser breaks are fantastic because the kids are energised. That's exactly what they are. They completely have a brain break. They reset." (Participant 1)

"We do use the energiser breaks now and then in the classroom. We try to take them out of class as much as possible for those brain breaks, because they get a little bit hyper in the classroom. But the energiser breaks are brilliant, and we might even do those with them outside as well, if we can." (Participant 2)

#### Modifications for Students with Disabilities

"The TREE framework is about bridging the gap. For lots of the iPLAY activities, there are modifications to be made to each game. It's about either using different sized balls or making the court smaller or having two groups instead of one big group. Sometimes they're given to you through the iPLAY website. But other times you as a teacher know your students well so you make your own modifications depending on the needs of the students." (Participant 1)

"The way that iPLAY has been set up really helps us teachers to come up with additional games and modified activities for the students to play. The equipment can be changed up, adjusted to suit the students and their individual needs and abilities as well." (Participant 2)

# Supplementary File 3: Comparison of Curricula for iPLAY versus iPLAY for Inclusion (iPLAY4i)

This document outlines the adaptations made to the original iPLAY curriculum to create iPLAY for Inclusion (iPLAY4i). The modifications were informed by evidence-based approaches for inclusive education, consultation with special education experts, and best practices in adapted physical education.

## Theoretical Framework Additions

The original iPLAY program was based primarily on the SAAFE framework (Supportive, Active, Autonomous, Fair, and Enjoyable). While iPLAY4i retains this foundation, it incorporates additional theoretical frameworks to better address the needs of students with intellectual disabilities:

| **Framework** | **Description** | **Rationale for Addition** |
| --- | --- | --- |
| TREE | Modifying **T**eaching style, **R**ules, **E**quipment, and **E**nvironment | Provides teachers with a structured approach to adapt activities for varied abilities while maintaining core learning objectives |
| Universal Design for Learning | Designing lessons with multiple means of engagement, representation, and action/expression | Shifts from post-hoc adaptation for individual students to embedding differentiation within lesson design for all students |
| Positive Behavior Support | Strength-based approach using positive reinforcement of desired behaviors | Addresses behavioral challenges common in PE settings through proactive, supportive strategies rather than punitive approaches |

##

## Module Structure Changes

### Added Module

Introduction to Intellectual Disability was added as a foundational module in iPLAY4i, positioned immediately after the Introductory Workshop. This module provides teachers with:

- Essential understanding of intellectual disability characteristics
- Methods for identifying student capabilities and limitations
- Strategies for gathering information about specific students' needs
- Frameworks for adapting physical activities appropriately

## Module Content Modifications

Modules were amended to remove content that was less relevant to make space for important new content. For example, "Differentiation & Empathy" in the original iPLAY was shortened to "Differentiation" in iPLAY4i. While empathy remains important, the module was refocused to provide more concrete differentiation strategies specific to intellectual disability rather than general communication of empathy.

In some cases, new videos replaced existing content to allow the course to focus on teachers of children with intellectual disability. For example, comparing the original and ‘inclusion’ version of the "Fair & Enjoyable" module:

### Content Removed

- Discussions of variety were removed as they were deemed less important
  - Feedback from special education experts indicated that excessive variety can be confusing for students with intellectual disability, who often benefit from consistency and clear routines

### Content Added

- "Using Support Staff" replaced the variety-focused content, reflecting the critical importance of effectively utilising School Learning Support Officers (SLSOs) and teaching assistants
- "Cues for Hopping" added specific instruction on teaching a fundamental movement skill often challenging for students with intellectual disability
- "NCCD Adaptation Opportunity" linked module content to the Nationally Consistent Collection of Data on School Students with Disability, helping teachers document adaptations for compliance purposes

These changes reflect a shift from general engagement strategies to practical, disability-specific adaptations and support utilisation.

## Key Content Adaptations Across All Modules

### Abbreviated Content

The following content areas were abbreviated in iPLAY4i compared to the original iPLAY:

1. ***Activity Variety and Complexity***
   - Original iPLAY emphasised varying activities to maintain engagement
   - iPLAY4i reduced this emphasis to create space for more differentiation
   - Rationale: Students with intellectual disability often benefit from predictable routines
2. ***Independent Decision-Making***
   - Original iPLAY encouraged student autonomy in activity design
   - iPLAY4i maintained choice but with more structured options
   - Rationale: Too many open-ended choices can overwhelm students with intellectual disability

### Expanded Content

Several areas received expansion in iPLAY4i:

1. ***Positive Behavior Support:*** Incorporated evidence-based strategies specific to physical activity contexts rather than general classroom management approaches.
2. ***Fundamental Movement Skill Instruction:*** Students with intellectual disabilities often require more systematic, explicit instruction to develop fundamental movement skills.
3. ***Support Staff Utilisation:*** Many students with intellectual disability have allocated support staff whose effective utilisation is valuable for successful inclusion.
4. ***TREE Framework Application:*** Systematically applied across all activities to provide teachers with a consistent framework for adaptations (Teaching style, Rules, Equipment, Environment).
5. ***Playground Inclusion Strategies:*** Added specific approaches for promoting social inclusion during unstructured play times where students with intellectual disabilities often experience isolation.
6. ***Sensory and Emotional Regulation:*** Included guidance on adapting established classroom coping strategies for physical activity settings.
